# Acute Sprint Interval Exercise Increases Both Cognitive Functions and Peripheral Neurotrophic Factors in Humans: The Possible Involvement of Lactate

**DOI:** 10.3389/fnins.2019.01455

**Published:** 2020-01-23

**Authors:** Sylwester Kujach, Robert Antoni Olek, Kyeongho Byun, Kazuya Suwabe, Emilia J. Sitek, Ewa Ziemann, Radosław Laskowski, Hideaki Soya

**Affiliations:** ^1^Department of Physiology, Faculty of Physical Education, Gdansk University of Physical Education and Sport, Gdańsk, Poland; ^2^Department of Athletics, Strength and Conditioning, Poznań University of Physical Education, Poznań, Poland; ^3^Sports Neuroscience Division, Advanced Research Initiative for Human High Performance, Faculty of Health and Sport Sciences, University of Tsukuba, Tsukuba, Japan; ^4^Division of Sport Science, Incheon National University, Incheon, South Korea; ^5^Neurological and Psychiatric Nursing Department, Faculty of Health Sciences, Medical University of Gdańsk, Gdańsk, Poland; ^6^Neurology Department, St. Adalbert’s Hospital, Poznań, Poland; ^7^Laboratory of Exercise Biochemistry and Neuroendocrinology, Faculty of Health and Sport Sciences, University of Tsukuba, Tsukuba, Japan

**Keywords:** sprint interval exercise, BDNF, IGF-1, VEGF, blood lactate, cognitive function

## Abstract

There is increasing attention to sprint interval exercise (SIE) training as a time-efficient exercise regime. Recent studies, including our own (Kujach et al., 2018), have shown that acute high-intensity intermittent exercise can improve cognitive function; however, the neurobiological mechanisms underlying the effect still remain unknown. We thus examined the effects of acute SIE on cognitive function by monitoring the peripheral levels of growth and neurotrophic factors as well as blood lactate (LA) as potential mechanisms. Thirty-six young males participated in the current study and were divided into two groups: SIE (*n* = 20; mean age: 21.0 ± 0.9 years) and resting control (CTR) (*n* = 16; mean age: 21.7 ± 1.3 years). The SIE session consisted of 5 min of warm-up exercise and six sets of 30 s of all-out cycling exercise followed by 4.5 min of rest on a cycling-ergometer. Blood samples to evaluate the changes of serum concentrations of brain-derived neurotrophic factor (BDNF), insulin-like growth factor-1 (IGF-1), vascular endothelial growth factor (VEGF), and blood LA were obtained at three time points: before, immediately after, and 60 min after each session. A Stroop task (ST) and trail making test (TMT) parts A and B were used to assess cognitive functions. Acute SIE shortened response times for both the ST and TMT A and B. Meanwhile, the peripheral levels of BDNF, IGF-1, and VEGF were significantly increased after an acute bout of SIE compared to those in CTR. In response to acute SIE, blood LA levels significantly increased and correlated with increased levels of BDNF, IGF-1, and VEGF. Furthermore, cognitive function and BDNF are found to be correlated. The current results suggest that SIE could have beneficial effects on cognitive functions with increased neuroprotective factors along with peripheral LA concentration in humans.

## Introduction

There is increasing attention to the beneficial effects of exercise on human cognition. Among various types of exercise, many studies have pointed out that moderate-intensity exercise has beneficial effects on cognitive abilities such as information processing or control of inhibition (Knaepen et al., 2010; Alves et al., 2012). However, these recommended aerobic exercise regimens require people to make a considerable time commitment, which is known as a limiting factor of physical activity in the modern era (Gibala and Little, 2010). Recently, high-intensity interval training (HIT) has become very popular among people as a time-efficient exercise regime (Gibala and Little, 2010; Astorino and Sheard, 2019). In addition, recent evidence suggests that HIT is more enjoyable than moderate-intensity endurance exercise (Bartlett et al., 2011; Little et al., 2011a). Since some reported that HIT could also be uncomfortable, causing effects such as active displeasure (i.e., distressed, upset) and lower enjoyment assessed with the Physical Activity Enjoyment Scale, especially for the sedentary population (Decker and Ekkekakis, 2017; Farias-Junior et al., 2019), various transferable forms of high-intensity intermittent exercise have been developed and it was revealed that these exercise regimes have beneficial effects not only on cardiovascular and metabolic adaptation but also on cognitive function (Little et al., 2010; Kujach et al., 2018). Sprint interval exercise (SIE) as a Wingate test-based modality consists of a number of supramaximal “all out” exercise bouts interspersed with recovery periods, achieving ∼20 min of activity in a single session (Burgomaster et al., 2005; Gibala and Little, 2010). This training protocol has been widely used and demonstrated to positively influence cardio-metabolic health parameters (Sloth et al., 2013). However, there is less evidence for how acute SIE improves cognitive functions and underlying neurobiological mechanisms.

Numerous animal and human studies have revealed that exercise enhances human cognition via exercise-enhanced neurotrophins and catecholamine production, which is known to mediate neural plasticity and energy metabolism in the brain (Kohut et al., 2006; Gomez-Pinilla and Hillman, 2013). Several neurochemicals, including brain-derived neurotrophic factor (BDNF), insulin-like growth factor-1 (IGF-1), and vascular endothelial growth factor (VEGF), are currently considered key proteins that mediate downstream effects of exercise on the brain and cognition (Cotman et al., 2007). As a single bout of physical exercise may lead to an increase in BDNF level (Ferris et al., 2007; Griffin et al., 2011), these exercise-increased neurotrophins may contribute to a reduction in mood disorders and to the protection and regeneration of various tissues resulting in increased cognitive performance in humans (Hansen et al., 2001; Rojas Vega et al., 2008; Zoladz and Pilc, 2010).

Interestingly, previous research has indicated that SIE facilitates muscle remodeling, which in turn may lead to the production of BDNF (Little et al., 2011b; Wrann et al., 2013; Phillips et al., 2014). In fact, high-intensity exercises and strength training both stimulate the secretion of IGF-1 (Bermon et al., 1999; Cassilhas et al., 2007), which is needed to transform pro-BDNF into BDNF in the central nervous system and can easily cross the blood–brain-barrier affecting neurogenesis and synaptic plasticity (Ding et al., 2006; Nishijima et al., 2010). In addition, recent animal studies have revealed that IGF-1 mediates exercise-induced angiogenesis, increased central BDNF, and VEGF production (Lopez-Lopez et al., 2004; Ding et al., 2006). Similar to IGF-1, peripheral VEGF also increases during exercise, in part mediating exercise-induced angiogenesis and neurogenesis (Lopez-Lopez et al., 2004). Circulating VEGF may promote neurogenesis and synaptic plasticity by stimulating neural stem cell proliferation and differentiation (Zacchigna et al., 2008; Ruiz de Almodovar et al., 2009) and also increases central endothelial cell and astrocytic productions of VEGF, BDNF, and IGF-1 (Zacchigna et al., 2008; Ruiz de Almodovar et al., 2009).

Moreover, SIE is related to the increase in glucose metabolism and lactate (LA) production, where glucose as well as LA are important energy sources for the human brain (van Hall et al., 2009; Tsukamoto et al., 2016; Hashimoto et al., 2018). Further, among various HIT protocols, SIE is well recognized as inducing important elevation in blood LA concentration (Wood et al., 2016; Olney et al., 2018). Glenn et al. (2015) found that peripherally produced LA is available as a cerebral energy supply after traumatic brain injury. Although the brain metabolism relies mainly on glucose while at rest, the cerebral consumption of glucose decreases during high intensity exercise, along with an increase in blood LA of consequence for the cerebral uptake (Kemppainen et al., 2005). Furthermore, improvement in executive functions may also be related with neuronal activation induced by high intensity intermittent exercise (Kujach et al., 2018). Neuronal activation is associated with an increase in energy requirement (Dalsgaard et al., 2002). Elevated peripheral LA concentration in response to intense exercise promotes the supply of LA as an energy substrate to meet acute neuronal energy requirements (Barros, 2013; Dienel, 2017). Thus, SIE modulating blood LA concentration and neuronal activation could affect cognitive function.

Although SIE may lead to progressive fatigue, causing excessive activation of the central nervous system and subsequent cognitive impairments (Chmura et al., 1994; Tomporowski, 2003), it might also induce the production of neurotrophins such as BDNF or IGF-1, positively affecting neurogenesis and synaptic plasticity in the brain (Lista and Sorrentino, 2010).

Given this evidence, we hypothesized that BDNF, IGF-1, and VEGF would increase in response to SIE and that this may be related to acute-exercise-induced cognitive benefits. Here, we have investigated whether acute SIE affects circulating neuroprotein concentrations, which may play a critical role in the enhancement of cognitive abilities.

## Materials and Methods

### Subjects

Thirty-six healthy, right-handed Polish-speaking male subjects participated in the study. All volunteers had normal vision (including color vision). No subject had a history of neurological, major medical, or psychiatric disorders, and none were taking medication at the time of measurement. Additionally, they were required to refrain from consumption of caffeine for 12 h prior to the testing session. All the subjects provided written informed consent prior to the study procedures. The participants were assigned to either the SIE group (SIE; *n* = 20, 21.0 ± 0.9 years) or the control group (CTR; *n* = 16, 21.7 ± 1.3 years) based on their age, weight, and physical fitness level (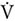
O_2max_). All the procedures were approved by the Bioethical Committee of the Regional Medical Society in Gdańsk.

### Study Design

**FIGURE 1:**
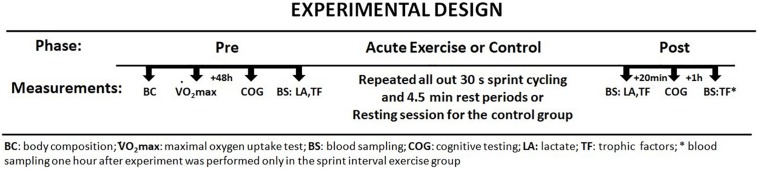
Study protocol – investigational timeline.

Participants visited the study site three times. One week before starting the experiment the subjects were asked to come to the laboratory for a familiarization session to learn about the testing procedures. Next, participants completed the same set of body composition, aerobic, and cognitive assessments before the main testing session. An overview of the experimental protocol is presented in Figure 1.

### Body Composition Measurements

Body mass (BM) and body composition were estimated using a multi-frequency impedance plethysmograph body composition analyzer (InBody 720, Biospace, South Korea). This analyzer accurately measures body water and body composition, including fat mass, free fat mass, skeletal muscle mass, and soft lean mass (Ziemann et al., 2013).

### Maximal Oxygen Uptake (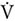
O_2max_) Test

To determine 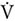
O_2max_, participants performed a graded cycle ergometry test on a mechanically braked cycle ergometer (884E Sprint Bike, Monark, Sweden). Subjects were allowed a 5-min warm-up period at an intensity of 1.5 W × kg^–1^ with a pedaling cadence of 60 rpm. Immediately after the warm-up the participants began 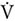
O_2max_ testing by cycling at increasingly difficult workloads in which resistance was increased by 25 W × min^–1^ until the participant reached the point of volitional exhaustion. 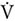
O_2max_ was determined when at least two of the following criteria were satisfied: (1) the respiratory exchange ratio (RER) exceeded 1.05, (2) achievement of 90% of age-predicted peak HR (220–age), and (3) an ratings of perceived exertion (RPE) of 19 or 20 (Suwabe et al., 2017). Breath-by-breath pulmonary gas exchange was measured (MetaMax 3B, Cortex, Germany) throughout the 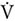
O_2max_ test; the O_2_ and CO_2_ analyzers were calibrated before each test using standard gases of known concentrations in accordance with manufacturer guidelines.

### SIE Sessions

Sprint interval exercise is a subcategory of interval exercise, involving “all out” supramaximal intensity (>100% 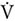
O_2max_) (Weston et al., 2014). The SIE sessions were performed on a mechanically braked cycle ergometer (884E Sprint Bike, Monark, Sweden). Exercise protocol started with a standard 5-min warm up at 1.5 Watts × kg^–1^ of BM. After the warmup, subjects performed the interval exercise, which included six sets of 30 s of “all out” sprint cycling exercise. Flywheel resistance equaled 0.075 kG × kg^–1^ of BM (i.e., Wingate test based) which corresponded to 7.5% of each individual’s BM and was applied on the onset of the SIE (Burgomaster et al., 2008). The interval rest periods between the 30-s bouts were 4 min and 30 s. The participants were instructed to accelerate until they reached their maximal pedaling rate and were verbally encouraged to maintain this pedaling cadence as long as possible throughout the SIE compilation. Only during the first few initial movements of each bout of the test was each participant allowed to pedal in a standing position; this was to help overcome the resistance and to quickly achieve the maximal pedaling rate. During the testing session, oxygen uptake was also monitored. For this purpose, a breath-by-breath pulmonary gas exchange method was used, where MetaMax 3B (Cortex, Germany) was applied. The O_2_ and CO_2_ analyzers were calibrated before each test using standard gases of known concentrations in accordance with manufacturer guidelines. Using MetaMax 3B, lung minute ventilation (VE L × min^–1^) and relative maximal oxygen uptake (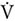
O_2max_ mL × min^–1^ × kg^–1^) were obtained. Heartrate (HR b × min^–1^) was monitored continuously via telemetry (S-625, Polar Electro-Oy, Finland). All training sessions were performed at similar times in the morning at least 2 h after breakfast.

### Cognitive Assessments

The assessment of cognitive functions was made using selected neuropsychological timed tasks. TMT A and B were used to measure working memory and the ST was adopted to assess executive functions. Since in meta-analyses, Lambourne and Tomporowski (2010) and Chang et al. (2012) suggest that more pronounced cognitive function facilitation could be observed 11–20 min following acute high-intensity exercise, each task was assessed before and 20 min after the SIE session for the SIE group, and before and 20 min after the resting session for the control group.

#### Stroop Test

The Polish paper version of the ST was used in this study. The ST consisted of 71 words written in colored ink. The participants’ task was to name the color of the font regardless of the word written. At the beginning of the test the meaning of each word was consistent with the color of the font in which it appeared (e.g., the word “blue” written in blue or “red” written in red; congruent) in order to activate the automatism of reading, and as the test progressed words written in incongruent fonts (e.g., the word “blue” written in green or “red” written in blue; incongruent) were randomly mixed in with the congruent words, requiring cognitive control in order to respond accurately. The numbers of “congruent” and “incongruent” stimuli were the same. The need to inhibit the automatic reading response, rather than to only name the color in which the word was printed, elicits a significant slowing in reaction time called the Stroop interference effect; thus, cognitive control is reflected in the time taken to execute the test and the number of errors. Consequently, the ST is a sensitive measure of cognitive inhibition and information processing speed (Stroop, 1935; Strauss et al., 2006).

#### Trail Making Test

The trail making test (TMT) is one of the most commonly used tests assessing working memory. This test includes two conditions (i.e., “A” and “B”), where the “A” condition reflects psychomotor speed and the “B” condition requires additional executive control needed to switch between number and letter sequences. TMT-A consists of drawing lines as quickly as possible to link consecutively numbered circles. In contrast, in TMT-B participants must connect circles while alternating between numbers and letters. Performance in both conditions is assessed based on the time (seconds) taken to complete each task (Chang and Etnier, 2009; Alves et al., 2012). The difference in time needed to complete part B and part A reflects the efficiency of the working memory, as both parts of the test are comparable in terms of visual search and motor requirements but do differ in cognitive complexity.

### Blood Sampling and Analysis

Samples were collected from the antecubital vein (*v. mediana cubiti*) between 8:00 and 10:00 a.m. to establish a baseline. Follow-up samples were taken before the warm-up, directly after the last (sixth) 30-s bout, and 1 h after the completion of all SIE bouts in order to evaluate serum concentrations of BDNF, IGF-1, VEGF, and cortisol. Samples for the CTR group were taken following the same timeline as those for the SIE group, except 1 h after the experiment.

The samples were centrifuged at 2000 × *g* for 10 min at 4°C. The separated serum samples were frozen and kept at −80 °C until later analysis. Serum BDNF, IGF-1, and VEGF were determined by enzyme immunoassay methods using commercial kits (R&D Systems, United States, catalog no. DBD00, DG100, DVE00). The average intra-assay CV was 8.0% for all proteins. Serum cortisol concentration was evaluated using a Demeditec (Germany) ELISA kit. Detection limits were 2.5 ng mL^–1^, and the intra-assay coefficient of variation for the kits was <7%. For blood LA analysis samples were collected from capillary blood taken from the finger as part of the baseline and after completion of last SIE bout. Immediately after collection, the blood was deproteinized by the addition of ice-cold 0.4 M perchloric acid. After being thoroughly mixed, the samples were centrifuged at 12,000 × *g* for 10 min. Blood LA was determined using a standard Randox (Crumlin, United Kingdom) kit based on the LA oxidase method (LC2389); assays were performed on a Cecil CE9200 spectrophotometer (Cambridge, United Kingdom).

### Statistical Analysis

The statistical calculations were performed using STATISTICA 13. The results are expressed as mean and standard deviation (SD), or standard error of mean (SEM). The normality of data distribution was established using the Shapiro–Wilk *W*-test. The level of significance was set as *p* = 0.05 for all of the analyses. Additionally, two-way analysis of variance (ANOVA) with repeated measures was used to investigate the significance of differences between groups and time. For peripheral neurotrophic factors concentration response to acute SIE a one-way repeated measures ANOVA was applied. Significant main effects were further analyzed with the Bonferroni or Tukey’s *post hoc* test. Changes (delta) in both groups were compared using an independent samples *t*-test or a Mann–Whitney *U* test, according to the data distribution. Correlations between variables were evaluated using the Spearman correlation coefficient for non-normally distributed data.

## Results

The study participants were physical education students who experience various forms of physical activity during the course of their studies (swimming, gymnastics, athletics, football, or basketball). Moreover, the results of body fat and 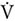
O_2max_ values demonstrate that the participants were physically active. All participants completed the study. The anthropometric and physical activity parameters are presented in Table 1. At baseline, there were no significant differences in basic anthropometric characteristics or in aerobic performance between groups (Table 1).

**TABLE 1:** Demographic and clinical characteristics.

Variable	SIE (*n* = 20)	CTR (*n* = 16)
Age (years)	21.0 ± 0.9	21.7 ± 1.3
Height (cm)	181.4 ± 6.4	183.3 ± 5.6
Weight (kg)	79.9 ± 7.9	83.1 ± 11.2
Fat (%)	13.0 ± 4.2	14.3 ± 6.4
Fat (kg)	10.5 ± 4.3	12.4 ± 6.5
SMM (kg)	39.9 ± 3.5	40.5 ± 4.1
BMI (kg × m^–2^)	24.2 ± 2.0	24.6 ± 2.5
TBW (kg)	50.8 ± 4.2	51.7 ± 5.0
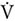 O_2max_ (mL × min^–1^ × kg^–1^)	48.6 ± 5.1	49.4 ± 6.2
Aerobic power (W)	285.7 ± 31.4	294.0 ± 35.6

*SIE, sprint interval exercise group; CTR, control group; values are mean ± SD expressed in absolute or relative values; Fat (%), fat percentage; Fat (kg), fat mass; SMM, skeletal muscle mass; BMI, body mass index; TBW, total body water; 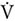
O_2max_, maximal oxygen uptake; aerobic power (W), maximal aerobic power achieved during 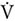
O_2max_ test.*

### Physiological Response to SIE

**FIGURE 2:**
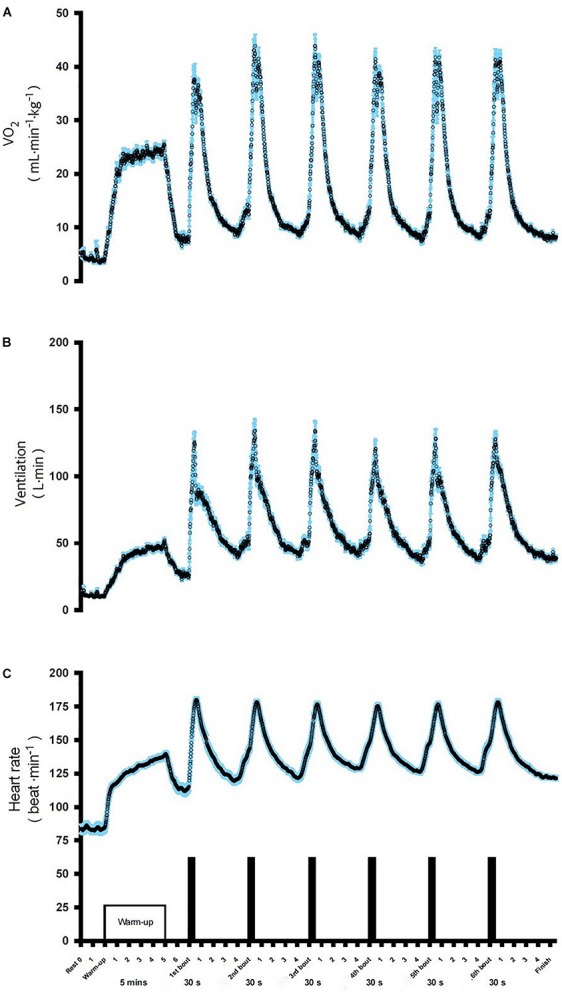
Oxygen consumption VO_2_ **(A)**, ventilation VE **(B)**, and heart rate HR **(C)**, during SIE. Data shown are means.

The evolution of VO_2_, VE, and HR during the six bouts of SIE are displayed in Figure 2. The obtained data indicate a significant increase in cardio-respiratory parameters compared to the baseline in the SIE group (Figure 2). The average 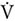
O_2_, VE, and HR responses to SIE correspond to ∼85, 90, and 90%, respectively, compared to the values reached in the maximal graded exercise test (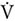
O_2max_ test). Moreover, the SIE group had RPE after acute SIE that averaged between 17–18 ± 2 (mean ± SD).

### Cognitive Performance in Stroop Test and Trail Making Test

**FIGURE 3:**
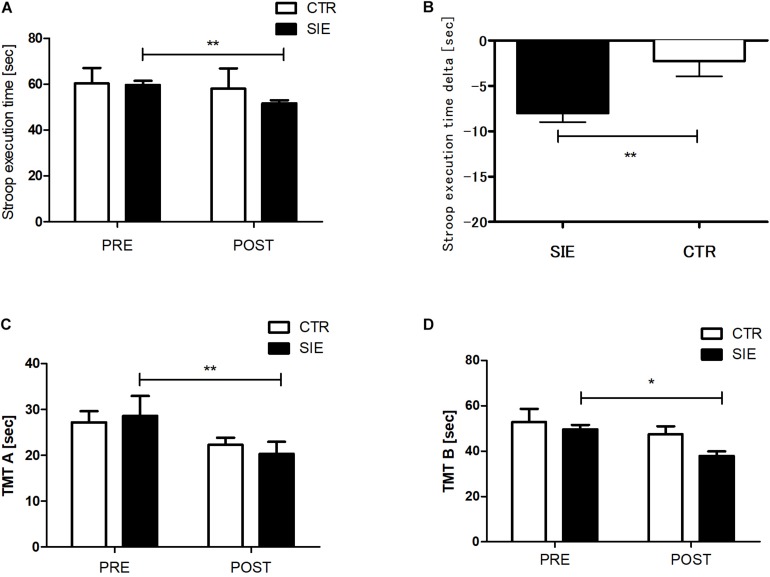
Stroop task **(A)** and trial making test, parts A and B **(C,D)** execution times. Contrast between SIE delta (post–pre) and CTR delta (post–pre) for Stroop task performance **(B)**. Values are means. Error bars indicate ± SEM (standard error of mean). *p < 0.05, **p < 0.001.

The analysis revealed no statistical differences between the SIE and CTR groups in ST performance (*p* = 0.85), TMT-A (*p* = 0.24), and TMT-B (*p* = 0.39) for the pre-sessions (Figure 3). There was significant interaction between group (SIE/CTR) and time (PRE/POST) factors when we performed a two-way ANOVA with repeated measures for ST execution time (*p* < 0.05; *F*_(__1_,_34_) = 9.45). Next, to examine the interaction, we calculated the difference of the degree of ST performance between post- and pre-sessions (delta), contrasted for both the SIE and CTR groups separately, and compared the difference between them. The delta ST performance difference was significantly more negative in the SIE than in the CTR group (*z* = 62.0; *p* < 0.001, Mann–Whitney *U* test) (Figure 3B).

Moreover there was a significant main effect of time in the TMT parts A and B (*p* < 0.05; *F*_(__1_,_68_) = 21.23 and *p* < 0.01; *F*_(__1_,_68_) = 6.42, respectively; two-way ANOVA), whereas neither main effect of group nor interaction of the factors was significant. *Post hoc* Tukey’s test analysis revealed a significant decrease in the both test version A (*p* < 0.01) and B (*p* < 0.05) execution time in the SIE group 20 min post-exercise (Figures 3C,D).

### The Response of Blood Lactate and Cortisol Levels Following Acute SIE

**FIGURE 4:**
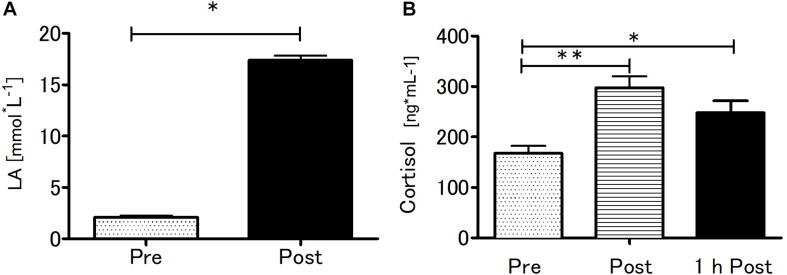
Blood lactate at baseline and after SIE **(A)** and serum cortisol concentrations pre, immediately post, and 1-h post-SIE **(B)** for the exercise group. Values are means. Error bars indicate ± SEM. *p < 0.05, **p < 0.001.

Blood LA concentration and serum cortisol concentration were significantly affected by SIE (Figure 4). The SIE resulted in a rapid increase in blood LA concentration (*t* = 35.5; *p* < 0.01). Acute SIE induced an almost twofold cortisol level increase (*p* < 0.01) sustained to 1 h post exercise (*p* < 0.05) relative to baseline: one-way repeated measures ANOVA with *post hoc* Bonferroni test results were *p* < 0.01; *F*_(__2_,_38_) = 18.51.

### Effect of Acute SIE on Peripheral Levels of BDNF, IGF-1, and VEGF

**FIGURE 5:**
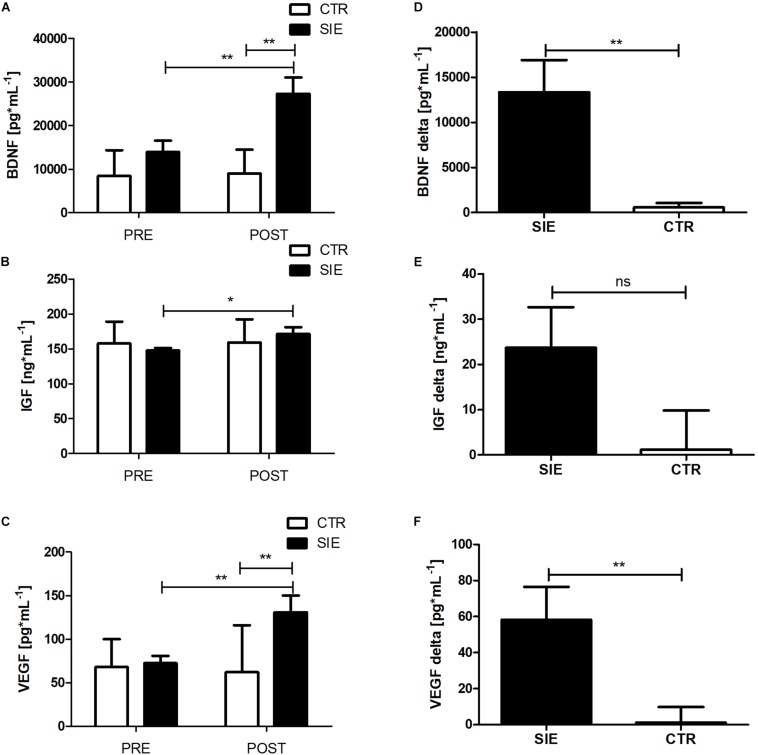
Effect of acute SIE on peripheral BDNF **(A)**, IGF-1 **(B)**, and VEGF **(C)** concentrations and contrast between SIE versus CTR deltas (post–pre) **(D–F)**. Values are means. Error bars indicate ± SEM. *p < 0.05; **p < 0.001; ns, non-significant.

Analyzing the effect of SIE on peripheral trophic factor concentration, a two-way ANOVA for repeated measures with *post hoc* Bonferroni test revealed a significant interaction (Group x Time) (*p* < 0.01; *F*_(__1_,_34_) = 10.18) in BDNF and VEGF (*p* < 0.01; *F*_(__1_,_34_) = 7.51) peripheral concentration (Figures 5A,C). Further, there was a significant main effect of time (*p* < 0.05; *F*_(__1_,_34_) = 3.87), whereas neither the main effect of group nor interaction of the factors was significant in peripheral IGF-1 concentration (Figure 5B). Moreover, statistically significant differences between the SIE and CTR groups in delta BDNF (*t* = 3.19; *p* < 0.01) and VEGF (*t* = 2.60; *p* < 0.01) concentrations were found (Figures 5D,F). Despite the increase in IGF-1 following SIE, statistical analysis did not show any significance effect versus the CTR group (*t* = 1.77; *p* = 0.08) (Figure 5E).

### The Response of Peripheral BDNF, IGF-1, and VEGF Following Acute SIE

**FIGURE 6:**
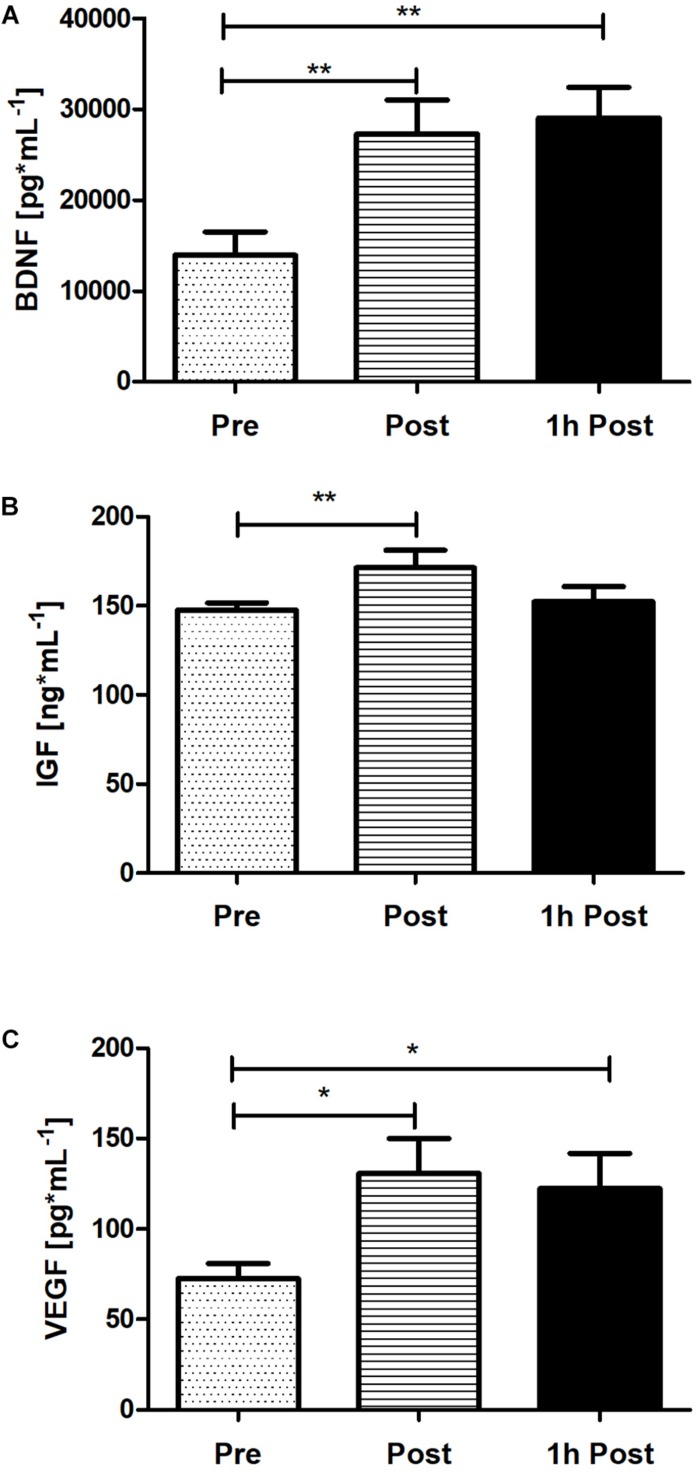
The response of serum BDNF **(A)**, IGF-1 **(B)**, and VEGF **(C)** concentration immediately post and 1 h following acute SIE. Values are means. Error bars indicate ± SEM. *p < 0.05, **p < 0.001.

To verify the peripheral BDNF, IGF-1, and VEGF concentration response to acute SIE a one-way repeated measures ANOVA and *post hoc* Bonferroni was applied. Acute SIE induced an increase in serum BDNF, IGF-1, and, VEGF concentration (Figure 6). There was a significant difference between time points in BDNF, IGF-1, and VEGF concentration (*p* < 0.01; *F*_(__2_,_38_) = 9.97, *p* < 0.01; *F*_(__2_,_38_) = 5.54, and *p* < 0.01; *F*_(__2_,_38_) = 5.23, respectively; one-way repeated measures ANOVA and *post hoc* Bonferroni test). *Post hoc* analysis revealed a significant increase in serum BDNF concentration immediately post-SIE (*p* < 0.01), sustained to 1 h post-SIE (*p* < 0.01), relative to baseline, as well as for VEGF concentration immediately post-SIE (*p* < 0.05), sustained to 1 h post-SIE (*p* < 0.05), relative to baseline. Acute SIE also significantly altered serum IGF-1 concentration immediately post exercise (*p* < 0.01) whereas at 1-h post-SIE serum IGF-1 concentration decreased and was not significantly different from the baseline. Changes in serum BDNF, IGF-1, and VEGF concentration after SIE are displayed in Figure 6.

**FIGURE 7:**
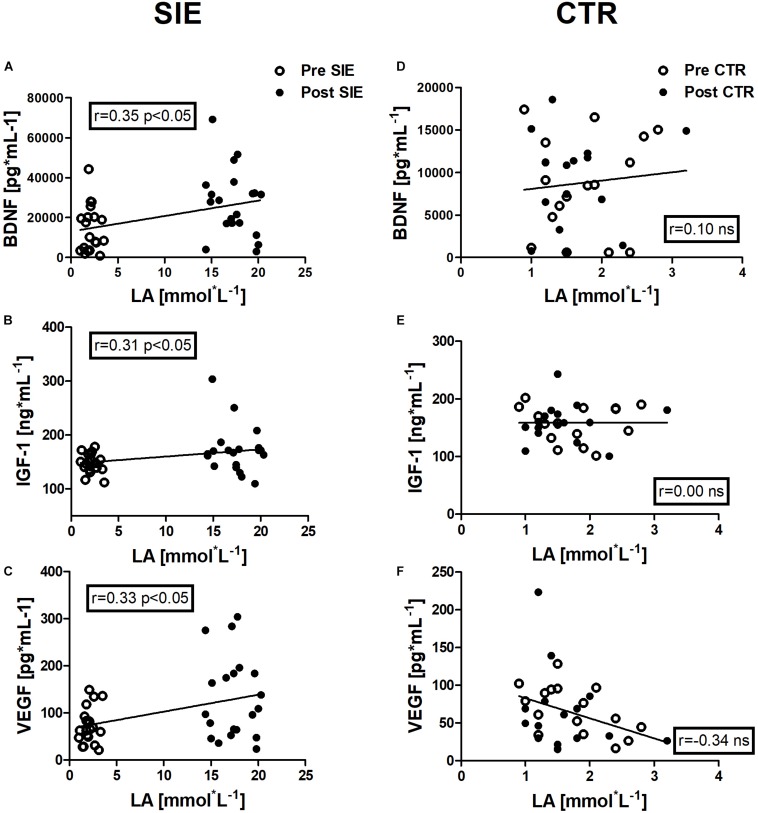
Association between LA and BDNF, IGF-1, and VEGF concentrations, of pre- and post-sessions in the SIE and CTR groups. Significant positive correlations between LA and BDNF concentration **(A)**, LA and IGF-1 concentration **(B)**, and LA and VEGF concentration **(C)** were found. No significant associations were observed in the concentration of neurotrophic factors and LA in the control group **(D–F)**. Data are presented as open circles: pre, and closed circles: post-SIE or CTR. ns, non-significant.

### Correlation Analyses

Positive correlations were identified between LA and BDNF concentration (*r* = 0.35, *p* < 0.05), between LA and IGF-1 concentration (*r* = 0.31, *p* < 0.05), and between LA and VEGF concentration (*r* = 0.33, *p* < 0.05) in the pre- and post-session in the SIE group.

**FIGURE 8:**
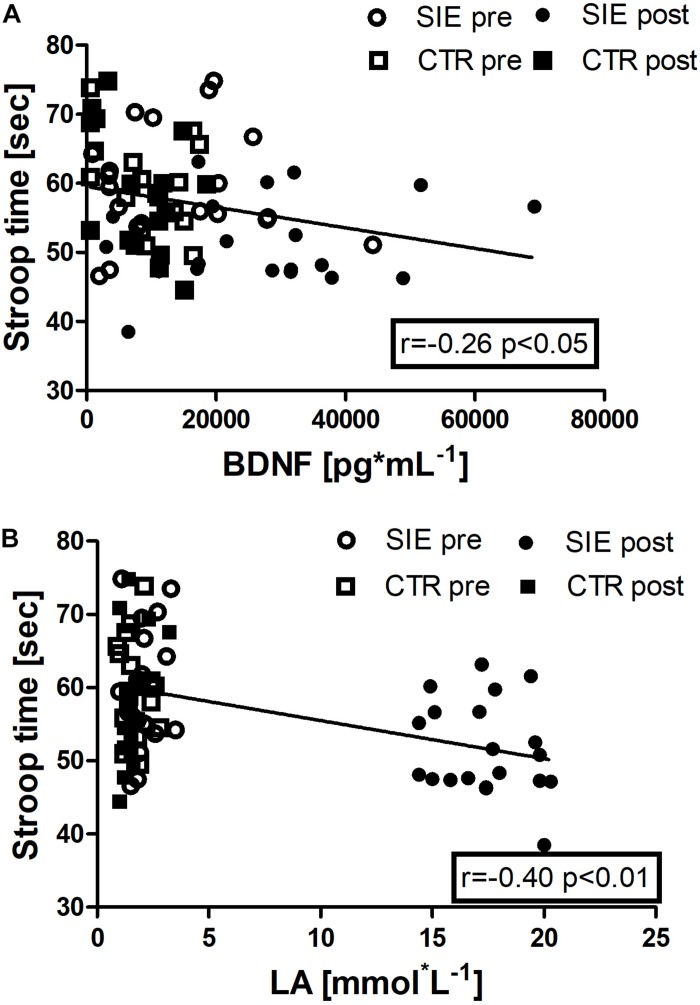
Association between Stroop execution time and BDNF concentration (*r* = –0.26, *p* < 0.05) **(A)**, and Stroop execution time and blood LA concentration (*r* = –0.40, *p* < 0.01) **(B)**. Data are presented as open circles: pre, and closed circles: post-SIE and open squares: pre, and closed squares: post-CTR.

In addition, we performed Spearman correlation analysis to examine the association between Stroop execution time and BDNF concentration (*r* = −0.26, *p* < 0.05), and a blood LA concentration (*r* = −0.40, *p* < 0.01) (Figures 8A,B).

## Discussion

In the present study, acute SIE led to an improvement in prefrontal-dependent cognitive performance. Also, SIE resulted in significant elevations of serum BDNF, IGF-1, and VEGF levels and these changes are accompanied by an increased peripheral LA concentration. These findings suggest that acute bouts of SIE are beneficial to improve cognitive performance 20 min following exercise in young adults.

We illustrated that acute SIE improved cognitive performance as indicated by shorter response times for the ST and faster completion time of the TMT parts A and B. These results are consistent with the findings of recent neuroimaging studies revealing improved cognitive performance on the ST and TMT A and B after acute aerobic exercise as well as with SIE, both of which are associated with changes of neural activation in the prefrontal cortex (Yanagisawa et al., 2010; Lee et al., 2014; Kujach et al., 2018). Therefore, we can postulate that the current SIE model is beneficial to the prefrontal-dependent cognitive functions in young, healthy adults.

Blood LA elevated through SIE is, potentially, a factor that leads to improved cognitive function together with SIE-induced neuroprotective protein levels. It has been demonstrated that glucose and LA are important energy sources not only in muscle, but also in the human brain (van Hall et al., 2009). At rest, the brain mainly relies on glucose, whereas during high-intensity exercise, glucose uptake significantly decreases with increased blood LA concentration (Kemppainen et al., 2005). In contrast, LA is used by the brain in order to compensate for the increased energy required to maintain neuronal activity during high-intensity exercise (Kemppainen et al., 2005; Weston et al., 2014). Recently, Hashimoto et al. (2018) showed that arterial LA and brain LA uptake (arterial–venous differences across the brain) increases after SIE, suggesting that systemic LA affects brain LA uptake and influences executive function after exercise (Weston et al., 2014). Moreover, we found a positive correlation between blood LA and neuro-supporting protein concentrations. Similarly, Ferris et al. (2007) found that the increased blood LA level induced by acute exercise correlated with blood BDNF level. Furthermore, it has been pointed out, that LA is the “missing exercise factor” inducing BDNF synthesis (El Hayek et al., 2019). Very recently, El Hayek et al. (2019) observed that LA modulates the redox status of neurons by altering the NAD^+^/NADH ratio which leads to SIRT1 activation and in turn engages the hippocampal PGC1-α/FNDC5 pathway to induce BDNF expression (Koltai et al., 2010; Wrann et al., 2013). Moreover LA released from exercising muscles mediates cerebral angiogenesis through the activation of the LA receptor HCAR1, a key regulator of VEGF (Morland et al., 2017). Additionally, LA induces IGF-1 mRNA expression via the somatotropic axis stimulation (Salgueiro et al., 2014). Since blood LA following SIE greatly increased in the present study, we cannot rule out that the improvement in cognitive functions resulted from the acceleration of cerebral LA metabolism along with neuroprotective proteins induction. However, various high-intensity interval training protocols could differently affect blood LA synthesis (Wood et al., 2016; Warr-di Piero et al., 2018). SIE is characterized by high LA production whereas longer protocols could rely more on aerobic metabolism, with lower LA synthesis (Astorino and Sheard, 2019). Therefore, interval training protocols could differently modulate the central nervous system activation and cognitive performance.

The present data also revealed that peripheral BDNF, IGF-1, and VEGF secretion are modulated by acute SIE. Recent studies proposed a hypothesis of the role of exercise-induced BDNF synthesis (Kemppainen et al., 2005; Wrann et al., 2013; Saucedo Marquez et al., 2015). Wrann et al. (2013) suggested that the activation of peroxisome proliferator-activated receptor-alfa coactivator (PGC)-1α is induced by exercise in skeletal muscle cells, and that it could activate FNDC5 gene expression, which is a positive regulator of BDNF levels in the brain, mainly in the hippocampus. Interestingly, an increase in PGC1-α has also been observed after exercise adopted in an interval exercise protocol (Little et al., 2011b). Moreover, Ferris et al. (2007) indicated that intensive exercise leads to greater increases in peripheral BDNF concentration than does low-intensity, continuous exercise, suggesting that exercise intensity could be a key factor. Saucedo Marquez et al. (2015) have speculated that skeletal muscle contractions during high-intensity exercise may trigger this biochemical pathway, inducing elevated BDNF levels in the brain. They revealed that the SIE protocol (10 × 1-min bouts at 90% of maximal work load, alternating with 1-min rest at 60 W for a total duration of 20 min) is a more effective and preferred intervention for elevating BDNF levels than traditional, continuous, moderate-intensity exercise (Saucedo Marquez et al., 2015). Furthermore, the high-intensity interval exercise protocol could also have induced optimal short bursts of oxidative stress and inflammation, leading to the activation of the prefrontal cortex a brain region involved in cognitive processing, including executive function (Gomez-Pinilla and Hillman, 2013). Our protocol was “all out” and characterized by a very high intensity which on the one hand stimulates dramatic BDNF increase and on the other hand could increase oxidative stress and inflammation to a greater extent. Thus, high-intensity exercises such as SIE, which is associated with increased inflammation and oxidative stress, will certainly be better tolerated by young active people in comparison to inactive persons or the elderly.

Moreover, it has been revealed that BDNF could modulate presynaptic neurotransmitter release and evoke excitatory postsynaptic currents via TrkB receptors, directly inducing neuronal depolarization (Kafitz et al., 1999; Jovanovic et al., 2000). Furthermore, we found a positive association between improved ST performance and BDNF concentration. Accordingly, other animal and human studies have shown significant, positive associations between serum BDNF and cortex BDNF (*r* = 0.81) at rest as well as elevated BDNF and improved cognitive performance in response to acute exercise (Karege et al., 2002; Kraus et al., 2004; Winter et al., 2007; Griffin et al., 2011). These findings suggest that acute SIE stimulates BDNF and may induce cognitive enhancement related to the above-mentioned changes in neural activation in the prefrontal cortex (Yanagisawa et al., 2010; Lee et al., 2014).

According to our initial hypothesis, acute SIE significantly increased peripheral IGF-1 concentration. Although IGF-1 concentration in the serum has been reported to increase following high-intensity exercise (Kraemer et al., 2004) other studies have reported a lack of post-exercise increase in peripheral IGF-1 concentration (Jahreis et al., 1989; Schiffer et al., 2009). Circulating IGF-1 is mainly derived from the liver, but its secretion is also found in the brain, skeletal muscle, or bones (Yakar et al., 1999; Dall et al., 2001). Therefore, it is assumed that for post-exercise IGF-1 concentration, peripheral and cerebral sources are partly responsible (Dall et al., 2001). Additionally, acute exercise stimulates the expression and release of liver IGF-1 and results in elevated brain uptake of IGF-1 (Carro et al., 2000). Also, peripheral IGF is necessary for exercise-induced hippocampal neurogenesis and for functional recovery after brain injury in rodents (Trejo et al., 2001; Duman et al., 2009). Similarly, to IGF-1 we found significant increase in VEGF concentrations. The effects of acute exercise on peripheral VEGF have produced conflicting results with some investigators finding an increase after acute exercise (Kraus et al., 2004; Wahl et al., 2011) and others finding no differences in VEGF concentration in healthy subjects after physical exercise (Landers-Ramos et al., 2014). Interestingly the level of VEGF in the hippocampus decreases with age, while post-exercise VEGF elevation could play an important therapeutic role increasing brain functions (Shetty et al., 2005). Peripherally circulating VEGF can affect neurogenesis and synaptic plasticity by inducing proliferation and differentiation of neural stem cells (Zacchigna et al., 2008; Ruiz de Almodovar et al., 2009). It appears that the IGF-1 as well as VEGF response are largely dependent on exercise intensity and a lack of consistency in study designs as far as exercise type, intensity, duration, and timing of blood sampling after exercise may explain some of these discordant findings (Griffin et al., 2011; Wahl et al., 2011; Skriver et al., 2014).

There is strong evidence from studies of both human and animal subjects that BDNF, IGF-1, and VEGF are important pathways by which chronic exercise modulates brain function (Cotman et al., 2007; Voss et al., 2013). Recent studies suggest that the three growth factors have an impact on functional brain connectivity in the medial and lateral temporal cortices (Voss et al., 2013). Moreover, BDNF may also influence functional connectivity by increasing synaptogenesis and dendritic spine density, therefore improving long-term potentiation (LTP) via increased synaptic plasticity (Schinder and Poo, 2000; Rex et al., 2006; Vaynman et al., 2006; Stranahan et al., 2007). In addition, periphery-produced IGF-1 and VEGF support exercise-induced neurogenesis and angiogenesis (Ding et al., 2006). Further, exercise-induced neurovascular adaptations in the hippocampus have been associated with cognitive function (Clark et al., 2009). Taken together, a growing number of evidence supports the importance of neurotrophic factors for synaptic plasticity and structural brain changes. However, these findings are mostly observed in chronic exercise intervention and were not replicated in the current study.

It has been revealed that exercise intensity may differently affect cognitive performance (Tomporowski, 2003; Kohut et al., 2006). Low- and moderate-intensity exercise improves cognition, whereas high-intensity exercise may lead to increased arousal resulting in impaired cognitive performance. For example, Chmura et al. (1994) observed gradual shortening in reaction time during a multiple-choice reaction task with an exercise intensity of up to ∼75% 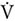
O_2__*peak*_, beyond which reaction time was rapidly impaired, suggesting that exercise intensity plays a role in the effects of acute exercise on some aspects of cognitive performance (Chmura et al., 1994). Moreover, prolonged high-intensity exercise exposure causes impairments in cognitive control and has neurotoxic effects in the human brain (Farias-Junior et al., 2019). In contrast, not only did our study not reveal any decrements in cognitive performance after an SIE session, but it also identified improvements in cognitive control and working memory. This is consistent with previous research adopting higher-intensity intermittent exercise, in which cognition parameters remained unaffected or even improved (Winter et al., 2007; Salgueiro et al., 2014). In addition, these findings are generally in agreement with other studies revealing that a single bout of HIT could improve executive performance and that this facilitation is sustained even for as long as 30 min (Yakar et al., 1999; Duman et al., 2009; Alves et al., 2014). The heterogeneity in the pattern of results of previous studies maybe due to differences in methodologies involving exercise mode and protocol, the participants’ fitness levels, cognitive task type, post-cognitive test timing, and other confounding factors (Chang et al., 2012).

It should also be noted that the high intensity of SIE could produce negative affective responses, thus making people unlikely to habitually perform the exercise (Hardcastle et al., 2014; Biddle and Batterham, 2015). We did not determine the mood state (enjoyment, pleasure, or affect) of our participants, so it is difficult to conclude whether they would like to repeat this kind of exercise in the future. However, Olney et al. (2018) demonstrated that healthy adults unaccustomed to interval training perceived high-intensity and SIE to be as enjoyable as time-consuming moderate-intensity continuous exercise. It is worth mentioning that the structure of interval training can be easily modified, allowing its application in people with diverse physical fitness levels. Therefore, there is a need for further research verifying the impact of various SIE protocols, especially since Townsend et al. (2017) showed that shorter sprint bouts (<30 s) in SIE produce greater pleasure among subjects. The proposed SIE can be successfully and safety applied by subjects with physical activity experience, such as physical education students or athletes. Collectively, we propose that even high intensity exercise, such as SIE, can be a fruitful and time-efficient intervention providing cognitive benefits to young, physically active people.

## Limitations

In this study we tested young, active, healthy men, making it difficult to generalize our results to the general population. Moreover, we did not control for BDNF polymorphism, which could also influence BDNF secretion in response to exercise.

## Conclusion

In summary, the current findings indicate that acute SIE enhances human cognitive function. The improvement in cognitive performance may result from the synthesis nor release of neuroprotective proteins modulated by high post-exercise blood LA concentration.

## Data Availability Statement

The datasets generated for this study are available on request to the corresponding author.

## Ethics Statement

The studies involving human participants were reviewed and approved by the Bioethical Committee of the Regional Medical Society in Gdańsk. The patients/participants provided their written informed consent to participate in this study.

## Author Contributions

SK, RO, and RL conceived of and designed the experiment. SK, RO, EZ, and RL collected the data. SK, KB, and ES performed the statistical analyses and interpreted the data. SK, RO, KB, ES, EZ, RL, and HS participated in drafting the article or revising it critically for important intellectual content. SK, RO, KB, KS, EZ, ES, RL, and HS approved the final version of the manuscript.

## Conflict of Interest

The authors declare that the research was conducted in the absence of any commercial or financial relationships that could be construed as a potential conflict of interest.
